# Vitamin D receptor *Taq* I polymorphism and the risk of prostate cancer: a meta-analysis

**DOI:** 10.18632/oncotarget.23606

**Published:** 2017-12-22

**Authors:** Shaosan Kang, Yansheng Zhao, Lei Wang, Jian Liu, Xi Chen, Xiaofeng Liu, Zhijie Shi, Weixing Gao, Fenghong Cao

**Affiliations:** ^1^ Department of Urology, North China University of Science and Technology Affiliated Hospital, Tangshan 063000, China; ^2^ Department of Imaging, KaiLuan General Hospital, Tangshan 063000, China; ^3^ Department of Surgery, LaoTing Traditional Chinese Medicine Hospital, Tangshan 063600, China; ^4^ Department of Urology, TangShan Gongren Hospital, Tangshan 063000, China

**Keywords:** Taq I, prostate cancer, vitamin D receptor, polymorphisms, meta-analysis

## Abstract

Numerous previous studies reported the association of Vitamin D receptor gene *Taq I*polymorphism with prostate cancer risk, however these results were controversial. In order to provide a relatively comprehensive description of this relationship, we conducted this meta-analysis by searching PubMed, Embase, and China National Knowledge Infrastructure. Finally, 36 studies with 8,423 cases and 8,887 controls were included. *Taq I* polymorphism was found to marginally increase the prostate cancer risk in recessive genetic model (tt/Tt vs. TT: Odds Ratio (OR) = 0.89, 95% Confidence Interval (CI) = 0.80–1.00, *p* = 0.05) and allele genetic model (t vs. T allele: OR = 0.91, 95% CI = 0.84–0.99, *p* = 0.003) in the overall analysis. Subgroup analyses showed that significant increased risk was found in Asians in homozygote model (tt vs. TT: OR = 0.63, 95% CI = 0.41–0.95, *p* = 0.029) and allele genetic model (t vs. T: OR = 0.78, 95% CI = 0.67–0.90, *p* = 0.002), and in the subgroup of population-based controls in all the genetic models. These results suggest that *Taq I*polymorphism might be a risk factor of prostate cancer risk, especially in Asians. It could be considered as a promising target to predict the prostate cancer risk for clinical practice.

## INTRODUCTION

Prostate cancer (PCa) is second-most commonly diagnosed malignancy in males, and thought to be one of the leading causes of cancer-related death around the world. In 2014, approximately 233,000 newly diagnosed cases and 30,000 PCa-related deaths was reported in America [1]. Furthermore, the global incidence is rising rapidly. By 2030, the number of new PCa and PCa-related deaths annually will climb to 1,853,391 and 544,209, respectively [2]. The etiology of PCa has remained unclear. Several factors are considered to significant increase the risk of PCa, including ethnicity, hormonal status, environment, diet, aging, and genetic factors [3].

Low serum levels of vitamin D might be one of the risk factors for PCa [4]. Laboratory investigation demonstrated that vitamin D inhibits the growth and differentiation of PCa cells, decreases the invasion, metabolism and angiogenesis of tumor cell. It can also promote tumor cell apoptosis [4]. In 2007, a clinical trial suggested that calcitriol, a kind of analogue of vitamin D can significantly improve patients’ survival rate by decreasing serum level of prostate special antigen (PSA) [5]. The antineoplastic effect of vitamin D is activated when binding to vitamin D receptor (*VDR*) [6]. 1,25-Dihydroxy vitamin D3(1,25(OH)_2_D_3_) is the hormonally active form of vitamin D. It binds to *VDR* and forms a heterodimer complex, which subsequently binds to the vitamin D response element and reduces the transcription levels of many genes that stimulating the cell growth and differentiation [7, 8].

Recently, the relationship of several single nucleotide polymorphisms (SNPs) of *VDR* gene and PCa risk has been the focus of research attention [8, 9]. *Taq I* polymorphism (rs731236) is one of the most widely-studied SNPs. It is a synonymous mutation located in exon 9 of *VDR* gene [10]. This mutation could reduce the mRNA stability and therefore decrease the mRNA levels of *VDR* gene [11]. Recently, some studies have suggested that *Taq I* variation might increase the susceptibility of PCa [12, 13]. However, these results are debatable and inconsistent in the effect of *Taq I* polymorphism on PCa risk. Numerous studies in favor of the association of *Taq I* polymorphisms and PCa risk [14–19]. Meanwhile, some studies disapprove of the relationship [20–22]. The difference might be due to under-power for individual study. Moreover, previous meta-analyses [10, 23, 24] seem to be outdated since new data appeared [17, 25–27]. Therefore, we conduct this meta-analysis to get more accurate results.

## RESULTS

### Characteristics of studies

**Figure 1:**
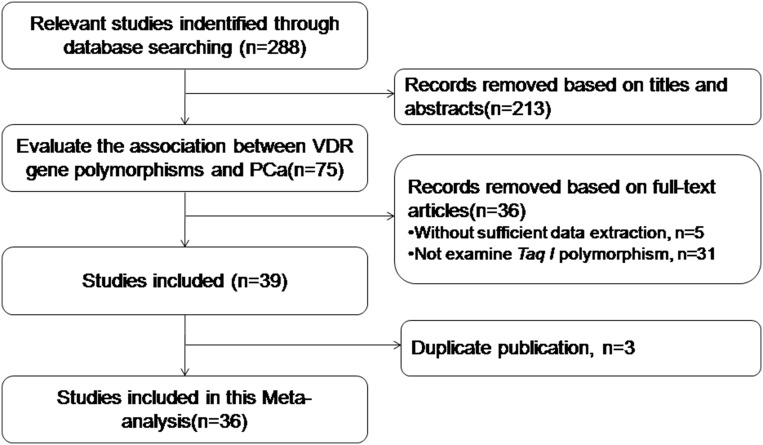
Study flowchart for the process of selecting the final 36 studies

We identified 288 potentially relevant studies following the retrieval strategy. Based on the inclusion criteria, 36 studies [3, 7, 9, 12, 14–19, 22, 25–49] between 1996 to 2017 were finally included (Figure 1). The number of cases and controls varied from 28 to 1,617, and 41 to 1,072, respectively (Table 1). The genotype distribution frequency in the control groups was consistent with Hardy-Weinberg equilibrium (HWE) for most studies, except for four studies [12, 19, 25, 49]. Each individual study scored more than 4 by Newcastle-Ottawa Scale (NOS), and was considered to be of high quality (Table 1). The percentages of tt, Tt and TT genotype in case group and control group were 11.9%, 40.4%, 47.7% and 12.1%, 41.3%, 46.6%, respectively in overall population.

**Table 1:** Characteristics and quality assessment of the studies included in this meta-analysis

Author	Year	Country	Ethnicity	Genotyping method	Sample size (cases/controls)	Source of Controls	HWE	NOS
Andersson	2006	Sweden	Caucasian	PCR-RFLP	137/176	PB	Y	6
Bai	2009	China	Asian	PCR-RFLP	122/130	HB	Y	6
Blazer	2000	USA	Caucasian	PCR-RFLP	77/183	PB	Y	6
Bodiwala	2004	UK	Caucasian	PCR-RFLP	368/243	BPH	N	6
Chaimuangraj	2006	Thailand	Asian	PCR-RFLP	28/30/44	HB/BPH	Y	5
Cicek	2006	USA	Mixed	PCR-RFLP	439/478	PB	Y	6
Correa-Cerro	1999	Germany/France	Caucasian	PCR-RFLP	106/95	HB	Y	6
Forrest	2005	UK	Caucasian	PCR-RFLP	262/444	HB	Y	6
Furuya	1999	Japan	Asian	PCR-RFLP	66/60	HB	Y	5
Gsur	2002	Austria	Caucasian	PCR-RFLP	190/190	BPH	Y	6
Habuchi	2000	Japan	Asian	PCR-RFLP	222/128/209	HB/BPH	Y	6
Hamasaki	2001	Japan	Asian	PCR-RFLP	115/133	HB	Y	6
Hamasaki	2002	Japan	Asian	PCR-RFLP	110/90/83	HB/BPH	Y	6
Holick	2007	USA	Caucasian	SNPlex	586/541	PB	Y	6
Holt	2009	USA	Caucasian	SNPlex	697/697	PB	Y	6
Hu	2014	China	Asian	TaqMan	108/242	PB	Y	6
Huang	2004	China	Asian	PCR-RFLP	160/205	PB	Y	6
Jingwi	2015	USA	African	TaqMan	306/251	PB	Y	6
John	2005	USA	African/Asian	TaqMan	424/436	PB	Y	6
Kambale	2017	India	Asian	PCR-RFLP	120/240	PB	N	5
Kibel	1998	USA	Mixed	PCR-RFLP	41/41	PB	Y	5
Luscombe	2001	UK	Caucasian	PCR-RFLP	209/154	BPH	Y	6
Ma	1998	USA	Caucasian	PCR-RFLP	354/589	HB	Y	7
Maistro	2004	Brazil	African	PCR-RFLP	165/200	HB	Y	6
Medeiros	2002	Portugal	Caucasian	PCR-RFLP	162/206	PB	Y	6
Oakley-Grivan	2004	USA	Mixed	PCR-RFLP	345/292	PB	Y	6
Oh	2013	Korea	Asian	IGGGS	272/173	BPH	Y	6
Onen	2008	Turkey	Caucasian	PCR-RFLP	133/157	PB	Y	6
Onsory	2008	India	Asian	PCR-RFLP	100/100	PB	Y	6
Rowland	2013	USA	Mixed	TaqMan	1617/1072	PB	Y	7
Suzuki	2003	Japan	Asian	PCR-RFLP	81/105	HB	Y	5
Tayeb	2003	UK	Caucasian	PCR-RFLP	21/379	BPH	Y	5
Tayeb	2004	UK	Caucasian	PCR-RFLP	28/56	BPH	Y	5
Taylor	1996	USA	Mixed	PCR-RFLP	108/170	BPH	Y	6
Watanabe	1999	Japan	Asian	PCR-RFLP	100/202	BPH	N	5
Yousaf	2014	Pakistani	Asian	PCR-RFLP	44/119	HB	N	5

Abbreviations: HWE, Hardy-Weinberg equilibrium; PB, population-based; HB, hospital-based; BPH, Benign Prostate Hyperplasia; RFLP, restriction fragment length polymorphism; NOS, Newcastle-Ottawa Scale.

### Pooled results

**Figure 2:**
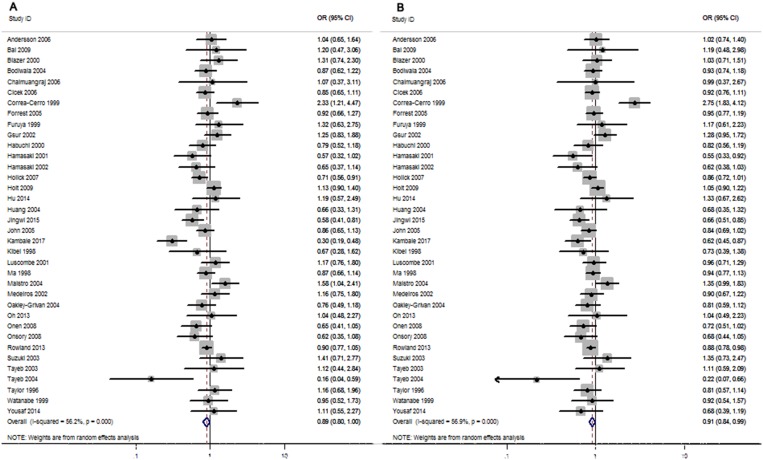
Forest plots to estimate the association of *VDR Taq I* polymorphism with PCa in the overall analysis (**A**) Dominant model (tt/Tt vs. TT). (**B**) Allelic frequency model (t vs. T allele).

As shown in Figure 2 and Table 2. Our results indicated that *Taq I* polymorphism marginally increase the PCa risk in the overall populations carrying TT genotype or T allele genotype (tt/Tt vs. TT: OR = 0.89, 95% CI = 0.80–1.00, *p* = 0.05; t vs. T allele: OR = 0.91, 95% CI = 0.84–0.99, *p* = 0.003), but not in other comparison models (tt vs. TT: OR = 0.86, 95% CI = 0.73−1.01, *p* = 0.069; Tt vs. TT: OR = 0.92, 95% CI = 0.81–1.10, *p* = 1.04; tt vs. TT/Tt: OR = 0.90, 95% CI = 0.76−1.06, *p* = 0.197) (Table 2).

**Table 2:** Results of the association between *Taq I* polymorphism and PCa risk in the whole population

Comparison	Studies	Overall effect			Heterogeneity		Publication bias	
		OR	Z-score	*p*-value	I^2^	*P*-value	Begg's test	Egger's test
tt vs TT	36	0.86 [0.73–1.01]	1.82	0.069	44.10%	0.004	0.382	0.363
Tt vs TT	36	0.92 [0.81–1.04]	1.35	0.176	60%	0.000	0.955	0.891
tt/Tt vs TT	36	0.89 [0.80–1.00]	1.96	0.05	56.20%	0.000	0.808	0.914
tt vs TT/Tt	36	0.90 [0.76–1.06]	1.29	0.197	54.20%	0.000	0.318	0.496
t vs T	36	0.91 [0.84–0.99]	2.18	0.03	56.90%	0.000	0.465	0.472

For the stratified analysis of different ethnicities, significantly increased risk was found in Asians in T allele genotype carriers (t vs. T: OR = 0.79, 95% CI = 0.68–0.91, *p* = 0.002) (Table 3 and Figure 2). However, when 15 studies performed in Caucasians and 3 studies in Africans were analyzed, no significant associations were found in any comparison models (Table 3).

**Table 3:** Results of the association between *Taq I* polymorphism and PCa risk in different ethnicities

Comparison	Studies	Overall effect			Heterogeneity		Publication bias	
		OR	Z-score	*p*-value	I^2^	*P*-value	Begg's test	Egger's test
**Asian**								
tt vs TT	14	0.63 [0.41–0.95]	2.18	0.029	0%	0.504	0.312	0.981
Tt vs TT	14	0.87 [0.63–1.21]	0.82	0.413	69%	0.000	0.033	0.022
tt/Tt vs TT	14	0.80 [0.63–1.03]	1.71	0.087	53%	0.010	0.055	0.023
tt vs TT/Tt	14	0.73 [0.38–1.39]	0.95	0.34	46%	0.046	0.243	0.414
t vs T	14	0.78 [0.67-0.90]	3.14	0.002	7%	0.373	0.033	0.026
**Caucasian**								
tt vs TT	15	0.99 [0.86–1.14]	0.08	0.935	56%	0.005	0.961	0.688
Tt vs TT	15	0.99 [0.85–1.16]	0.08	0.933	50%	0.014	0.961	0.878
tt/Tt vs TT	15	1.00 [0.85–1.17]	0.03	0.974	55%	0.05	0.961	0.762
tt vs TT/Tt	15	1.01 [0.81–1.26]	0.08	0.938	62%	0.001	0.656	0.913
t vs T	15	1.01 [0.89–1.14]	0.12	0.905	67%	0.000	0.729	0.884
**African**								
tt vs TT	3	0.96 [0.45–2.08]	0.1	0.922	72%	0.027	0.602	0.603
Tt vs TT	3	0.94 [0.51–1.72]	0.22	0.829	82%	0.004	0.602	0.632
tt/Tt vs TT	3	0.94 [0.50–1.78]	0.18	0.855	85%	0.001	0.602	0.581
tt vs TT/Tt	3	0.96 [0.59–1.56]	0.17	0.86	40%	0.189	0.602	0.515
t vs T	3	0.96 [0.61–1.52]	0.18	0.86	85%	0.002	0.602	0.597

**Figure 3:**
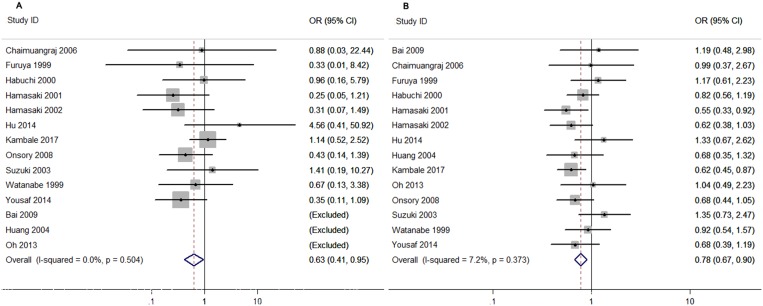
Forest plots to estimate the association of *VDR Taq I* polymorphism with PCa in Asians (**A**) Homozygote model (tt vs. TT). (**B**) Allelic frequency model (t vs. T allele).

*Taq I* polymorphism could significantly increase PCa risk in the subgroup of population-based controls when patients carrying TT genotype or T allele genotype in all the genetic models (tt vs. TT: OR = 0.83, 95% CI = 0.73–0.94, *p* = 0.004; Tt vs. TT: OR = 0.83, 95% CI = 0.69–1.00, *p* = 0.049; tt/Tt vs. TT: OR = 0.82, 95% CI = 0.70–0.96, *p* = 0.016; tt vs. TT/Tt: OR = 0.88, 95% CI = 0.78–0.98, *p* = 0.023; t vs. T allele: OR = 0.89, 95% CI = 0.84–0.95, *p* = 0.000) (Table 4 and Figure 3). Meanwhile, results for the subgroups of hospital-based and BPH controls revealed no significantly increased risk (Table 4).

**Table 4:** Results of the association between *Taq I* polymorphism and PCa risk in different source of controls

Comparison	Studies	Overall effect			Heterogeneity		Publication bias	
		OR	Z-score	*p*-value	I^2^	*P*-value	Begg's test	Egger's test
**Population-based**								
tt vs TT	16	0.83 [0.73–0.94]	2.98	0.003	2%	0.429	0.882	0.843
Tt vs TT	16	0.83 [0.69–1.00]	1.97	0.049	73%	0.000	0.471	0.437
tt/Tt vs TT	16	0.82 [0.70–0.96]	2.41	0.016	68%	0.000	0.719	0.419
tt vs TT/Tt	16	0.88 [0.78–0.98]	2.28	0.023	27%	0.155	0.961	0.862
t vs T	16	0.89 [0.84–0.95]	3.89	0.000	39%	0.057	0.418	0.297
**Hospital-based**								
tt vs TT	12	0.90 [0.51–1.59]	0.37	0.710	70%	0.000	0.815	0.481
Tt vs TT	12	1.02 [0.81–1.30]	0.19	0.851	50%	0.025	0.411	0.406
tt/Tt vs TT	12	0.99 [0.78–1.27]	0.07	0.946	57%	0.008	0.681	0.752
tt vs TT/Tt	12	0.89 [0.51–1.54]	0.42	0.675	72%	0.000	0.484	0.390
t vs T	12	0.97 [0.76–1.25]	0.21	0.832	77%	0.000	0.681	0.767
**BPH**								
tt vs TT	11	0.90 [0.68–1.19]	0.75	0.451	20%	0.267	0.677	0.476
Tt vs TT	11	1.01 [0.85–1.20]	0.11	0.911	25%	0.208	0.938	0.715
tt/Tt vs TT	11	0.98 [0.83–1.16]	0.22	0.823	17%	0.282	0.586	0.586
tt vs TT/Tt	11	0.85 [0.66–1.10]	1.23	0.217	43%	0.083	0.677	0.585
t vs T	11	0.95 [0.85–1.08]	0.76	0.447	24%	0.219	0.586	0.501

Studies were stratified into TaqMan, PCR-RFLP, and SNPlex groups by genotyping methods. No significant association was found in almost subgroups, except TaqMan group in tt vs. TT/Tt comparison (Table 5). The pooled outcome indicated that the genotyping methods included in these studies are both available and did not alter the outcomes.

**Table 5:** Results of the association between *Taq I* polymorphism and PCa risk in different genotyping method

Comparison	Studies	Overall effect			Heterogeneity		Publication bias	
		OR	Z-score	*p*-value	I^2^	*P*-value	Begg's test	Egger's test
**PCR-RFLP**								
tt vs TT	29	0.88 [0.71–1.01]	1.13	0.258	46%	0.006	0.393	0.283
Tt vs TT	29	0.94 [0.80–1.10]	0.77	0.441	62%	0.000	0.970	0.702
tt/Tt vs TT	29	0.91 [0.79–1.05]	1.31	0.19	57%	0.000	0.851	0.995
tt vs TT/Tt	29	0.90 [0.71–1.13]	0.93	0.35	58%	0.000	0.307	0.277
t vs T	29	0.92 [0.82–1.03]	1.51	0.13	59%	0.000	0.476	0.424
**TaqMan**								
tt vs TT	4	0.71 [0.53–0.94]	2.38	0.017	32%	0.219	1.000	0.794
Tt vs TT	4	0.85 [0.69–1.05]	1.50	0.134	44%	0.147	0.174	0.691
tt/Tt vs TT	4	0.82 [0.65–1.02]	1.79	0.074	53%	0.093	0.497	0.812
tt vs TT/Tt	4	0.77 [0.64–0.93]	2.68	0.007	3%	0.378	1.000	0.618
t vs T	4	0.83 [0.71–0.97]	2.36	0.018	49%	0.117	1.000	0.995
**SNPlex**								
tt vs TT	3	0.95 [0.75–1.20]	0.43	0.669	0%	0.322	0.317	-
Tt vs TT	3	0.91 [0.60–1.39]	0.43	0.664	78%	0.010	0.602	0.999
tt/Tt vs TT	3	0.92 [0.64–1.33]	0.45	0.656	74%	0.022	0.602	0.997
tt vs TT/Tt	3	1.01 [0.81–1.25]	0.05	0.961	0%	0.783	0.317	-
t vs T	3	0.96 [0.82–1.23]	0.52	0.603	37%	0.203	0.602	0.987

**Figure 4:**
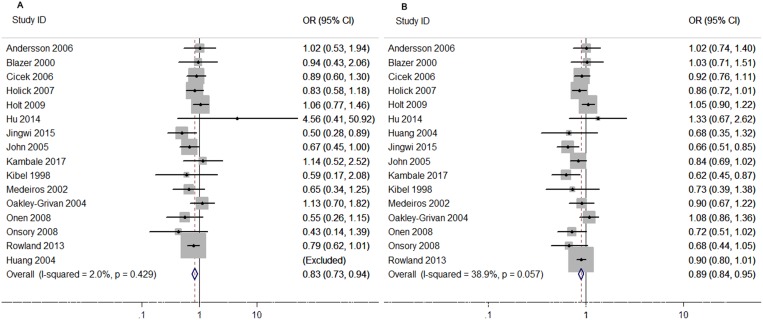
Forest plots to estimate the association of *VDR Taq I* polymorphism with PCa in the subgroup of population-Based controls (**A**) Homozygote model (tt vs. TT). (**B**) Allelic frequency model (t vs. T allele).

As shown in Figure 4 and Table 6, we also performed a stratified analysis based on the clinical stages by Gleason Score to describe the relationship in more detail. The pooled results from 9 studies for advanced tumor group and 8 studies for localized tumor group did not reveal any association of *Taq I* polymorphism with the PCa risk in various genetic models. When 4 studies deviated from HWE in the controls were excluded, similar results were obtained (The results were not given).

**Table 6:** Results of the association between *Taq I* polymorphism and PCa risk in different tumor stage

Comparison	Studies	Overall effect			Heterogeneity		Publication bias	
		OR	Z-score	*p*-value	I^2^	*P*-value	Begg's test	Egger's test
**Advanced**								
tt vs TT	9	0.87 [0.66–1.14]	1.02	0.307	23%	0.243	0.621	0.763
Tt vs TT	9	0.85 [0.65–1.11]	1.18	0.237	53%	0.030	0.404	0.357
tt/Tt vs TT	9	0.84 [0.64–1.10]	1.28	0.200	59%	0.012	0.532	0.347
tt vs TT/Tt	9	0.92 [0.69–1.22]	0.59	0.552	34%	0.155	0.621	0.686
t vs T	9	0.88 [0.70–1.10]	1.14	0.252	66%	0.003	0.211	0.301
**Localized**								
tt vs TT	8	0.63 [0.27–1.45]	1.10	0.273	85%	0.000	0.453	0.966
Tt vs TT	8	0.90 [0.66–1.24]	0.63	0.531	61%	0.013	0.458	0.901
tt/Tt vs TT	8	0.84 [0.56–1.27]	0.83	0.406	79%	0.000	0.458	0.933
tt vs TT/Tt	8	0.66 [0.35–1.22]	1.33	0.182	76%	0.000	0.652	0.891
tvs T	8	0.84 [0.69–1.01]	0.95	0.344	86%	0.000	0.621	0.903

### Heterogeneity

Significant between-study heterogeneity was detected in the overall analysis for all the comparison models (tt vs. TT: *p* = 0.004, I^2^ = 44%) Tt vs. TT: *p* = 0.000, I^2^ = 60%; tt/Tt vs. TT: *p* = 0.000, I^2^ = 56%; t vs. TT/Tt: *p* = 0.000, I^2^ = 54%; and t vs. T allele: *p* = 0.000, I^2^ = 57%) (Table 2). Therefore, random-effects estimates would be more suitable for data analysis. In the subgroup analyses of ethnicity, no heterogeneity was detected in homozygosis genetic model (*p* = 0.504, I^2^ = 0%) or allele-frequency model (*p* = 0.373, I^2^ = 7%) (Table 3). Similarly, subgroup analysis of population-based controls reported no heterogeneity in homozygosis model, recessive model or allele-frequency model (Table 4). Fix-effect model was applied in these comparison models.

### Publication bias and sensitivity analysis

**Figure 5:**
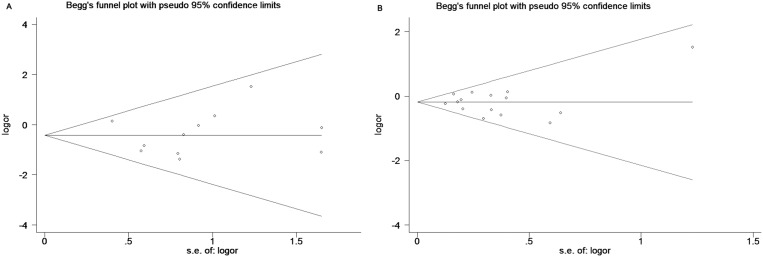
Begg's funnel plots to examine publication bias for reported comparisons of *VDR* gene *Taq I* polymorphism for the homozygote in (A) Subgroup of Asians (**B**) Subgroup of Population-Based controls.

**Figure 6:**
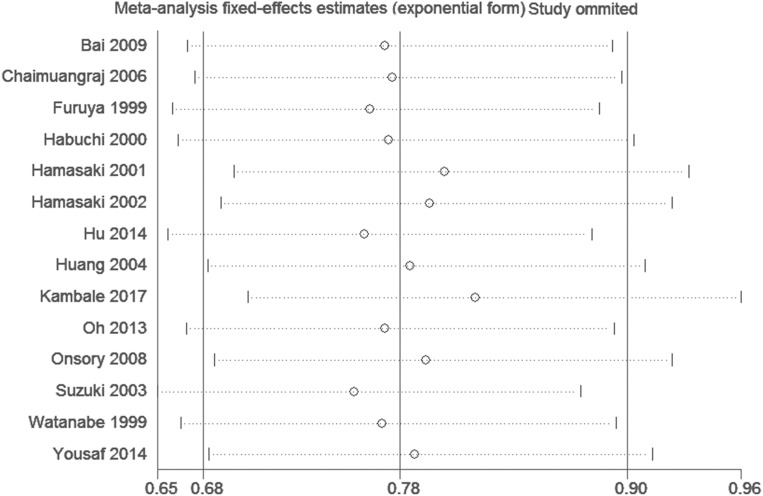
Sensitivity analysis of the comparison in Allelic frequency model (t vs. T allele) in Asians

As shown in Figure 5, funnel plots did not reveal any obvious asymmetry. Moreover, the Egger's test also showed that there was no publication bias in the overall analysis (Table 2) and almost the subgroup analyses (Table 3-6). Sensitivity analyses suggested that the pooled results had not changed significantly by omitting each individual study from all the analyses (Figure 6).

## DISCUSSION

In recent years, polymorphism of *VDR* gene has drawn great attention, because more and more studies have shown that the mutations of *VDR* gene were related to the PCa risk [14–19]. However, these results have been disputable [20–22]. Previous meta-analyses were reported by Yin et al. in 2009, Fei et al. in 2016 and Liu et al. in 2017 [10, 23, 24], in which the number of included studies was 23, 27 and 8, respectively. However, some new data was reported, which is not consistent with the results of the former three studies [17, 25–27]. 8,423 cases and 8,867 controls were included in our analysis from 36 independent studies. The cases included were much more than the previous meta-analyses. Therefore, our results might be more convincing and stringent.

Our meta-analysis showed that *Taq I* polymorphism might increase the PCa risk in overall population in recessive genetic model and allele-frequency genetic model. It is not consistent with the results of previous report by Liu et al. [10]. But for the stratified analysis of ethnicity, significant increased risk was found to be associated with *Taq I* polymorphism in Asians, which is consistent with the results of the report of Fei et al [24]. Ethnicity is an important biological factor for the decline of VDR function [50]. The difference in outcome among ethnicities might result from racial backgrounds and geographic discrepancies [51]. In addition, different dietary patterns could also contribute to the difference [52]. Our results suggested that the *Taq I* variation might be one of the valuable biomarkers for predicting the susceptibility of PCa. Further studies of Caucasian and African are required.

For the subgroup analysis by the source of controls, increased risk of PCa was found to be associated with *Taq I* polymorphism in population-based controls in all the comparisons. Possibly some sick population were included in the groups of HBP or hospital-based controls, these groups were special and could not represent all the population [53]. Therefore, the results of these groups might be lack of credibility. Our results revealed some discrepancies between the genotyping methods. It suggested that *Taq I* polymorphism in the subgroup of TaqMan, *Taq I* was associated with PCa risk, which may be the cause of heterogeneity. According to a report in 2004, clinical tumor stage of PCa would be accelerated by *VDR* gene polymorphism [54]. Hence, we performed a subgroup analysis by clinical stage. Our results indicated no association between *Taq I* polymorphism and susceptibility of PCa, which were different from the previous meta-analyses [24].

Although the between-study heterogeneity was detected, sensitivity analysis did not reveal any significant change in our results by omitting the studies contribute to the heterogeneity. It suggested that our results were credible and statistically robust.

Some limitations should be acknowledged. First, several studies with too little number of patients were included in our analysis, they may introduce potential bias. Second, our results were based on unadjusted parameters, a more accurate analysis are needed, in which some related parameters should be included to adjust the outcome, including age, diet, and other important lifestyle factors.

In conclusion, our meta-analysis might be the largest meta-analysis to estimate the association of *VDR* gene *Taq I* polymorphism with the risk of PCa. Marginally increase of PCa risk was found to be related with *Taq I* polymorphism in overall population, especially in Asians and in population-based controls subgroup. In the future, large and well-designed researches are required to demonstrate the increased effect of *Taq I* polymorphism on PCa risk.

## MATERIALS AND METHODS

### Literature and search strategy

The PubMed, Embase, and Chinese National Knowledge Infrastructure (CNKI) database searches were carried out for all the eligible papers. The following search terms were included: “*VDR*/vitamin D receptor”, “prostate cancer/tumor/carcinoma” and “polymorphism/mutation/variant”. The literature search was updated to August, 2017. In addition, manually searching for the additional studies was conducted according to the references of the original and review reports.

### Study selection

Retrieved studies were deemed eligible provided that they met all of the following criteria: (a) studies on human beings; (b) in a case-control or nested case-control design; (c) investigated the relationship of *Taq I* polymorphism with PCa risk; (d) distribution of genotype frequency for cases and controls could be obtained or calculated; (e) and received more than 4 points in the NOS, which was considered to be high quality; (f) the difference of baseline characters and clinical information was not significant between PCa patients and controls.

### Data extraction

The studies meeting the inclusion criteria were read carefully by two investigators independently (Yansheng Zhao and Xiaofeng Liu). We collected the following information: author, year, country, ethnicity, genotyping methods, source of controls, sample size, and genotype and allele frequencies. The subjects were divided into different subgroups: Asians, Africans and Caucasians for ethnicity; hospital-based, population-based, and Benign Prostate Hyperplasia (BPH) for the source of controls; TaqMan, PCR-RFLP and SNPlex for genotyping method. The clinical stages were categorized as localized group (Gleason < 7) and advanced group (Gleason ≥ 7). In order to reach consensus on all of the items, any disagreement was resolved by a third reviewer (Lei Wang).

### Statistical analysis

A χ^2^-test based on the Q statistic was conducted to evaluate the heterogeneity. The between-study heterogeneity was considered to be significant when I^2^ > 50% and *p* < 0.05, and the random effects model was used to combine values from studies [55]. Otherwise, for homogeneous studies, the fixed effects model was chosen.

The pooled odds ratios (ORs) together with its 95% confidence intervals (95% CIs) were calculated to evaluate the strength of the association. The statistical significance of ORs was determined with Z-test. To get a more reasonable result, five genetic models were adopted in our analysis: homozygote model (tt vs. TT), heterozygous model (Tt vs. TT), dominant model (tt vs. TT/Tt), recessive model (tt/Tt vs. TT) and allele genetic model (t vs. T).

To assess the potential publication bias, Begg's Funnel plot was generated based on the analysis result and database size. The more asymmetry the funnel plot looked, the more publication bias was introduced. Meanwhile, Egger's test was also performed for further investigation. For the Egger's test, the significance level was set as *p* value < 0.05. Moreover, HWE of controls was recalculated with the goodness-of-fit χ^2^-test, *P* values of > 0.05 was considered as significant equilibrium.

For each outcome, we also performed subgroup analyses according to ethnicity, source of controls, genotyping method and clinic stages. Sensitivity analysis was performed to assess the stability of pooled results.

All analyses were performed using STATA package version 12.0 (Stata Corp, College Station, TX, USA). Two-sided *P* values of < 0.05 was considered to be statistically significant.
